# Electrochemical Detection of C-Reactive Protein in Human Serum Based on Self-Assembled Monolayer-Modified Interdigitated Wave-Shaped Electrode

**DOI:** 10.3390/s19245560

**Published:** 2019-12-16

**Authors:** Somasekhar R. Chinnadayyala, Jinsoo Park, Young Hyo Kim, Seong Hye Choi, Sang-Myung Lee, Won Woo Cho, Ga-Yeon Lee, Jae-Chul Pyun, Sungbo Cho

**Affiliations:** 1 Department of Electronic Engineering, Gachon University, Seongnam-si, Gyeonggi-do, Incheon 13120, Korea; ssreddy@gachon.ac.kr; 2 Gachon Advanced Institute for Health Science & Technology, Gachon University, Incheon 21999, Korea; jspark88@gc.gachon.ac.kr; 3 Department of Otorhinolaryngology-Head and Neck Surgery, School of Medicine, Inha University, Incheon 22332, Korea; inhaorl@inha.ac.kr; 4 Department of Neurology, School of Medicine, Inha University, Incheon 22332, Korea; seonghye@inha.ac.kr; 5 Department of Chemical Engineering, Kangwon National University, Chuncheon 25341, Korea; sangmyung@kangwon.ac.kr; 6 Cantis Inc., Ansan-si, Gyeonggi-do 15588, Korea; wwcho@cantis.co.kr; 7 Department of Materials Science and Engineering, Yonsei University, Seoul 03772, Korea; gayeon@yonsei.ac.kr (G.-Y.L.); jcpyun@yonsei.ac.kr (J.-C.P.)

**Keywords:** interdigitated wave-shaped microelectrode array, self-assembled monolayer, C-reactive protein, immunosensor, atomic force microscopy, impedance spectroscopy

## Abstract

An electrochemical capacitance immunosensor based on an interdigitated wave-shaped micro electrode array (IDWµE) for direct and label-free detection of C-reactive protein (CRP) was reported. A self-assembled monolayer (SAM) of dithiobis (succinimidyl propionate) (DTSP) was used to modify the electrode array for antibody immobilization. The SAM functionalized electrode array was characterized morphologically by atomic force microscopy (AFM) and energy dispersive X-ray spectroscopy (EDX). The nature of gold-sulfur interactions on SAM-treated electrode array was probed by X-ray photoelectron spectroscopy (XPS). The covalent linking of anti-CRP-antibodies onto the SAM modified electrode array was characterized morphologically through AFM, and electrochemically through cyclic voltammetry (CV) and electrochemical impedance spectroscopy (EIS). The application of phosphate-buffered saline (PBS) and human serum (HS) samples containing different concentrations of CRP in the electrode array caused changes in the electrode interfacial capacitance upon CRP binding. CRP concentrations in PBS and HS were determined quantitatively by measuring the change in capacitance (ΔC) through EIS. The electrode immobilized with anti-CRP-antibodies showed an increase in ΔC with the addition of CRP concentrations over a range of 0.01–10,000 ng mL^−1^. The electrode showed detection limits of 0.025 ng mL^−1^ and 0.23 ng mL^−1^ (S/N = 3) in PBS and HS, respectively. The biosensor showed a good reproducibility (relative standard deviation (RSD), 1.70%), repeatability (RSD, 1.95%), and adequate selectivity in presence of interferents towards CRP detection. The sensor also exhibited a significant storage stability of 2 weeks at 4 °C in 1× PBS.

## 1. Introduction

C-reactive protein (CRP; 118 kDa) is a homopentameric classical acute phase inflammatory protein [1]. Expression of CRP increases during inflammatory autoimmune diseases such as rheumatoid arthritis, some cardiovascular diseases, and infections [2]. Many studies have reported the role of CRP as a potential biomarker for coronary heart disease, and likely as a direct contributor in vascular inflammation [3]. The reference level of CRP in the blood of healthy individuals is below 3 μg mL^−1^ and changes between 0 and 1 μg mL^−1^ (low risk), 1–3 μg mL^−1^ (intermediate risk), and 3–10 μg mL^−1^ (high risk) are indicative of potential cardiovascular events [4]. Pepys and Hirschfield reported that minute changes between 0.1 and 10 μg mL^−1^ are strongly associated with predictions of future coronary events, while larger increases are related to chronic inflammatory diseases, such as arthritis [5]. Therefore, detection of very low levels of CRP is needed for early detection of cardiovascular risk and inflammatory events. Several methods have been reported for CRP detection, including a CRP enzyme-linked immunosorbent assay (ELISA), surface plasmon resonance [6], piezoelectric cantilevers [7], quartz crystal microbalances [8], and electrochemical analysis [9], as well as nephelometric, turbidimetric, and luminometric methods [10]. However, many efforts are still ongoing to improve the detection limit, dynamic range, reliability, cost, and measurement speed of such analytical methods.

To quantify very low concentrations of CRP in clinical laboratories, an ELISA protocol with a limit of detection as low as 1.0 µg mL^−1^ is usually applied [11]. The detection limit is improved to 1.0 ng L^−1^ if the ELISA is incorporated with beads [12]. Nevertheless, the detection limit needs to be improved further for clinical applications. Electrochemical ELISA assays and surface plasma resonance [13] based sensors have demonstrated CRP detection at the μg and sub-μg levels. However, CRP detection at the pg and sub-pg levels, which would facilitate the early detection of cardiovascular events, is not well demonstrated. Therefore, the sensitive quantification of CRP abundance using rapid, reliable, and precise analytical methods in pharmaceutical research and clinical diagnosis has become an important objective.

Recently, label-free electrochemical impedimetric immunosensing techniques have been reported using microdisc ITO (Indium tin oxide) electrodes and interdigitated gold electrode arrays for selective and ultrasensitive detection of tumor necrosis factor-α, human insulin, and CRP at the µg and sub-µg mL^−1^ levels [14,15,16]. In addition, developments in the microfabrication industry have enabled the fabrication of miniaturized electrochemical sensing platforms to improve the detection limits of immunosensors [17]. Efforts are being made to develop CRP electrochemical sensing systems for point-of-care testing applications and personalized health monitoring. Owing to developments in the microfabrication industry, it is now possible to fabricate microelectrodes in different configurations that are highly sensitive and provide very low detection limits and dynamic ranges. The fabrication of a simple interdigitated micro electrode arrays in wave shapes for electrochemical CRP quantification with low-cost manufacturing has made the present electrochemical immunosensing study competitive with other immunosensing techniques.

SAMs provide the simplest methods to produce ultrathin, reproducible, oriented, and ordered monolayers that can retain the bioactivity of functionalized macromolecules. SAM coatings on gold surfaces using carboxylic acid (–COOH) functional groups containing thiols such as 11-mercaptoundecanoic [18], dithiobutyric [19], 3-mercaptoproponoic, and thioctic acids [20] are needed to activate N-hydroxysuccinimide(NHS)/(N-(3-Dimethylaminopropyl)-N′-ethylcarbodiimide hydrochloride (EDC) before use under stringent reaction conditions (e.g., strict acidic conditions), which increases the complexity of SAM preparation. However, dithiobis (succinimidyl propionate; DTSP) can be easily used without additional activation steps compared with other SAMs by forming covalent amide bonds between amino groups of protein lysine residues and reactive succinimidyl group of DTSP [21]. The mild reaction conditions and short self-assembling time of DTSP (2 h) increases the accuracy and practical utility of microelectrode surface functionalization.

Herein, we fabricated a label-free electrochemical capacitance immunosensor by functionalization of electrode arrays using SAMs. The detection antibodies were covalently linked onto functionalized SAM surfaces through amide cross-linking. As a prototype, the electrode arrays were functionalized with DTSP-SAM, and human anti-CRP-antibody was selected as the model immobilizing antibody to detect recombinant human CRP protein. The CRP antigen was detected by probing the interfacial properties of electrodes, (ΔC; capacitance change) using electrochemical impedance spectroscopy. The fabricated sensor with dimensions (16 × 63 mm) has the capability to function as a portable electrochemical immunosensing system to detect CRP for point-of-care applications. The BSA/anti-CRP-antibodies/DTSP/IDWµE array immunosensor detected CRP at sub-pg levels within 10 min. In addition, the DTSP-modified microelectrode array surfaces provided a suitable sensing platform for CRP sensing excluding SAM activation steps (EDC)/(NHS) and retained the bioactivity of the immobilized antibodies.

## 2. Materials and Methods

### 2.1. Materials

Recombinant human CRP and anti-CRP-antibodies were procured from AbCam (Cambridge, UK). Dithiobis (succinimidyl propionate), sodium borohydride (NaBH_4_), Bovine serum albumin (BSA), human serum (HS), potassium ferrocyanide (K_4_Fe(CN)_6_), potassium ferricyanide (K_3_Fe(CN)_6_), and polydimethylsiloxane (PDMS) were procured from Sigma-Aldrich (St. Louis, MO, USA). Tween-20 was purchased from Amresco (Solon, OH, USA). 1× PBS comprising 0.05% Tween-20, and Phosphate-buffered saline (10× PBS, pH 7.4) were purchased from Tech and Innovation (Gangwon, Korea). All solutions were prepared with deionized water of resistivity 18.2 MΩ cm supplied by Purescience (Jungwon, Korea). All the chemicals used in the assay procedures were of analytical grade and are used as received without further purification.

### 2.2. Instruments

Atomic force microscopy (AFM) images were obtained using an ambient air scanning probe microscope (XE–100 Park Systems, Korea) operated in a typical non-contact mode with scanning at a rate of 0.5 Hz. The scanning region was approximately 1 × 1 µm^2^ with a resolution of 0.05 nm. Images were analyzed using XEP software for root mean square roughness, height differences, and skewness (asymmetry of the height distribution). A scanning electron microscope (HITACHI S-4700, Japan) provided with energy dispersive X-ray analyzer (HORIBA EX 250) was used to study the elemental composition of the electrode arrays before and after SAM treatment. To study the gold-sulfur interactions and elemental electronic structure, X-ray photoelectron spectrometer (XPS) provided with an Al-K_α_ monochromatic X-ray source from Thermo Electron Corporation (Waltham, MA, USA) was used. The high vacuum pressure in the analysis chamber during XPS scans was maintained below 10^−9^ Torr. Survey spectra (data not shown) were recorded first with 200 eV pass energy, 1 eV steps, and a dwell time of 50 ms. The sample spot size was approximately 4 × 4 µm^2^, and the takeoff was maintained at 45°. The S(2p) and C(1s) region scans were determined with typical bandwidths of 15–20 eV, pass energies of 50 eV, step sizes of 1 eV, and dwell times of 50 ms. The obtained XPS spectra were deconvoluted using standard Gaussian curve fit with Shirley background subtraction [22]. Electrochemical measurements were recorded using a potentiostat from IVIUM technologies (CompactStat, Eindhoven, The Netherlands). The voltammograms were determined in 5 mM [Fe(CN)_6_]^3−^/^4−^) (1:1; *v*/*v*) in 1× PBS (pH 7.4, 25 °C), at a scan rate of 50 mV s^−1^ in a potential window of +0.5 V to −0.5 V in a three-electrode system consisting of working electrode (bare IDWµE or modified IDWµE array), a counter electrode (platinum coil), and a reference electrode (Ag/AgCl electrode in NaCl). Impedance spectroscopy data of the bare IDWµE or modified IDWµE arrays was also measured using potentiostat from IVIUM technologies, in 1× PBS (pH 7.4, 25 °C) using a two electrode configuration. The EIS spectra were obtained by applying an A.C perturbation voltage with root mean square value of 50 mV in 1 Hz–100,000 Hz frequency range with five points per decade. The obtained impedance spectra were fitted to a simplified equivalent circuit using a non-linear curve fitting software (ZView 3.2c; Scribner Associates Inc., Southern Pines, NC, USA).

### 2.3. Fabrication of Interdigitated Wave-Shaped Microelectrode Array

For the capacitive detection of CRP, an interdigitated wave shaped micro electrode array (IDWµE) was fabricated on a glass slide substrate (63 × 16 × 1.1 mm) using microelectromechanical system (MEMS) technology. A conductive adhesion metal layer of Ti, having a thickness of 25 nm and an Au electrode metal layer, having a thickness of 50 nm were deposited successively using electron beam evaporation. The spacing and width of the interdigitated fingers were both 30 µm and were patterned by a photolithographic and chemical wet etching process. The patterned electrode array consisted of a sensing region, transmission lines, and terminal pads. Next, a PDMS chamber (d = 8 mm) prepared was attached to the sensing area of the electrode array for the formation of SAMs, and to preserve the solvents for affinity reactions and impedance spectra measurement (Figure S1).

### 2.4. Functionalization of Electrode Arrays with DTSP

The fabricated IDWµE arrays were initially cleaned by successive sonication in acetone, ethanol, and DI water for 2 min, followed by rinsing with milli Q water, and purged under low stream of N_2_ gas to remove any impure materials bound on the electrode surface. DTSP was functionalized on the sensing area of the electrode arrays by immersion in 2 mg mL^−1^ DTSP solution prepared in acetone for 2 h. Before SAM functionalization, DTSP solution was first reduced with 10 mg mL^−1^ aqueous NaBH_4_ for 10 min at room temperature (25 °C) [23,24,25]. Subsequently, the SAM-functionalized electrode arrays were rinsed with acetone followed by DI water to remove free thiol moieties. An additional washing step was performed by sonicating the substrate in fresh acetone for 5 min to remove H-bonded thiol moieties. Subsequently, the electrodes were washed with acetone and DI water for 2 min and blown dry under low stream of N_2_ gas.

### 2.5. Affinity Immunoassay Protocol

**Figure 1:**
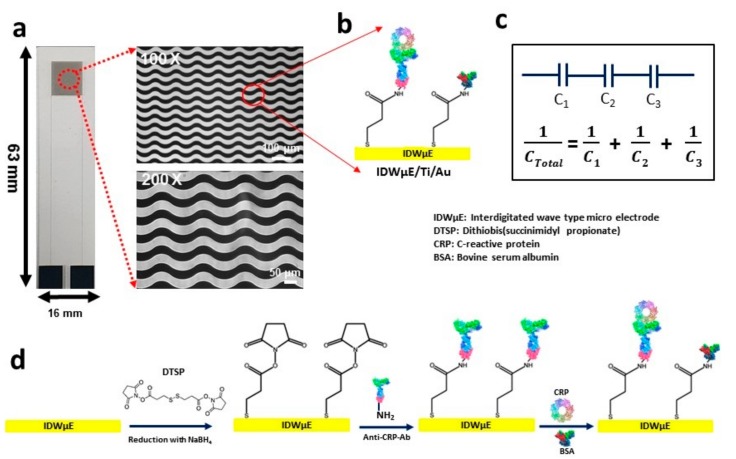
Microscopic image of the IDWµE fabricated on a glass slide with dimensions of 16 mm × 63 mm × 1.1 mm. (**a**) The electrode array microscopic images at Low magnifications (100×) and high magnifications (200×) showing a 30 µm width for finger and spacing, respectively. (**b**) A schematic illustration of the DTSP functionalization and immobilization of anti-CRP-antibodies onto the IDWµE array. (**c**) The underlying working principle of the CRP immunosensor based on quantifying the total capacitance of the sensor after sequential formation of SAM, anti-CRP antibody, BSA, and CRP layers; (where C1 = C_SAM_, C2 = C_anti-CRP-Ab_, C3 = C_CRP_). (**d**) The sequential surface modification steps of the IDWµE array for immunosensing of CRP.

The DTSP-functionalized electrode array was incubated with 20 μL of anti-CRP-antibodies (50 μg mL^−1^) and placed in a humid chamber for 1 h to avoid drying of the electrode array surface during the binding process. Lysine‒NH_2_ groups of anti-CRP-antibodies and the reactive succinimidyl group of the DTSP-SAM formed stable covalent amide bonds during the binding process on the electrode surface. The anti-CRP antibody/DTSP/IDWµE arrays that formed were washed with 1× PBS containing 0.05% Tween-20 for 2 min to remove unbound anti-CRP-antibodies. To avoid nonspecific binding, BSA (1 mg mL^−1^) in 1× PBS, pH 7.4, was used to form BSA/anti-CRP-antibody/DTSP/IDWµE arrays. Finally, the electrodes thus formed (anti-CRP-antibodies/DTSP/IDWµEs) were washed with 1× PBS containing Tween-20 (0.05%) to remove unbound BSA molecules. Different quantities of CRP were mixed into 10 mM of PBS (pH 7.4) and HS to obtain final concentrations of 0.01, 0.1, 1, 10, 100, 1000, 5000, and 10,000 ng mL^−1^. The functionalization of DTSP-SAM, covalent crosslinking of anti-CRP-antibodies onto the electrode array chips, the affinity sensor electrode surface modification steps, and the underlying detection principle are shown in Figure 1.

### 2.6. Preparation of HS Samples

HS was diluted 1:100 using PBS, pH 7.4, in order to circumvent matrix effects. A range of CRP concentrations (0.01–10,000 ng mL^−1^) in HS samples was prepared and applied to the electrode array for incubation (10 min) in a humid chamber to react with the available CRP.

## 3. Results and Discussion

### 3.1. Characterization of Modified Electrode Arrays

#### 3.1.1. Chemical Composition of the Modified Electrode Arrays

**Figure 2:**
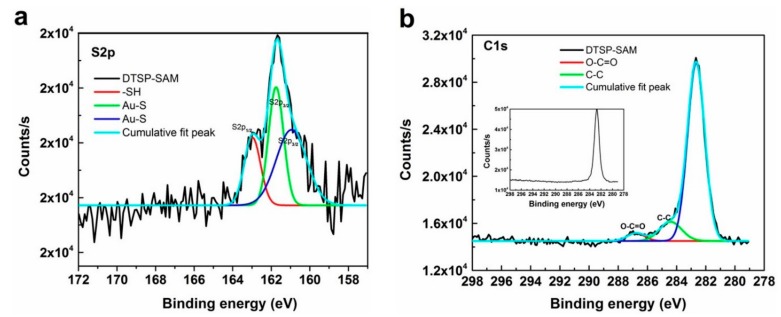
X-ray photoelectron spectroscopy spectra (XPS) of DTSP-SAM on IDWµE: (**a**) S2p and C1s (**b**) bands.

To confirm DTSP‒SAM functionalization on the electrode array surface, EDX measurements were first conducted to study the elemental composition of the microelectrode array before and after SAM treatment. Figure S2 shows EDX images of the bare microelectrode array (a) and DTSP-SAM/IDWµE array (b) surfaces in the 0–10 keV range. The bare electrode array spacing area displays O and Si as the major elements, with Al and Ca as trace elements, while the working area of the electrode arrays displays Au as the major element and Si, Ti, and Ca as trace elements. After DTSP‒SAM treatment, the working area of the electrode arrays had Au as the major element and S, Si, Ti, C, and Ca as trace elements. Where the elements Au, Si, and Ti were from the electrode array surface and additional elements S and C on electrode surfaces rose due to SAM functionalization on the electrode array. In addition, to probe the nature of SAM–Au interactions, and focusing on the contributions of the sulfur and carbon atoms, S2p and C1s, XPS bands are of prime importance to understand the degree of bonding to the electrode array surface, as shown in Figure 2a,b. For the, S2p deconvoluted spectra, three distinct components were detected; the binding energies (BE) at 161.5 eV and 161.0 eV can be ascribed to Au-bonded sulfur atoms [25], whereas the signal at 163.0 eV is typical of non-bonded or weakly bound sulfur atoms [26]. Figure 2b, displays a high-resolution deconvoluted carbon 1s (C-1s) spectrum of the DTSP functionalized Au electrode array. A BE of 284.4 eV was assigned to the alkyl chains (C‒C) in the DTSP-SAM [27]. Furthermore, the functionalization of the SAM on Au electrode arrays was probed by the additional signal detected at 287.0 eV, which is typical of the carboxyl group (O‒C=O) ([28]) of SAM functionalized on Au electrode arrays. The above two signals were not detected in the high resolution C1s spectrum of the bare Au electrode array (inset of Figure 2b).

#### 3.1.2. Topography of Modified Electrode Arrays

**Figure 3:**
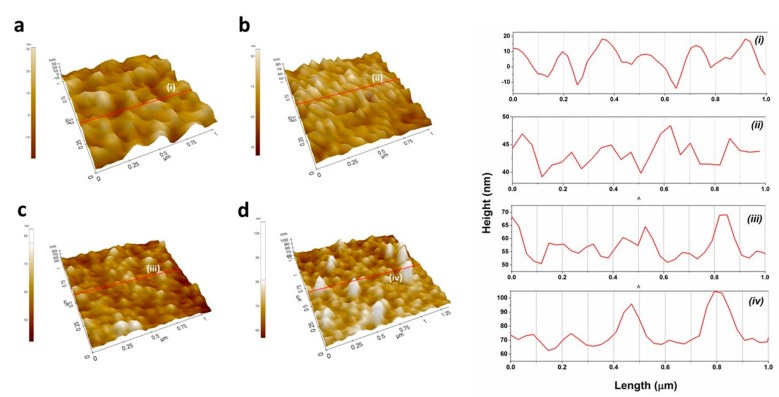
AFM topographical images of DTSP/IDWµE (**a**), DTSP/IDWµE (**b**), anti-CRP-antibody/DTSP/IDWµE (**c**), and CRP/BSA/anti-CRP-antibody/DTSP/IDWµE arrays (**d**) scanned at a rate of 0.5 Hz with topographic profile.

To study morphological changes of the electrode surface after major steps of electrode modification, AFM topological images were recorded. The bare plane gold electrode array surface exhibited a polycrystalline surface with an average roughness of 8.097 ± 0.42 nm (n = 10) (Figure 3a). The electrode surface was transformed to a smooth surface topography in comparison to bare gold surfaces after DTSP-SAM functionalization, with an average roughness of 4.078 ± 0.1 nm (n = 10) (Figure 3b).

The electrode surface was changed to a globular structure after modification with anti-CRP-antibodies, suggesting the successful cross-linking of antibody on the surface, with an increase in electrode roughness to 5.08 ± 0.37 nm (n = 10) (Figure 3c). Finally, with the addition of CRP target analyte, the electrode surface showed a clear protruding topography across the electrode surface. The protruding topography was due to stable antigen-antibody complex formation on electrode surfaces, with increased surface roughness of 6.1 ± 0.2 nm (n = 10) (Figure 3d).

### 3.2. Optimization of Experimental Conditions

**Figure 4:**
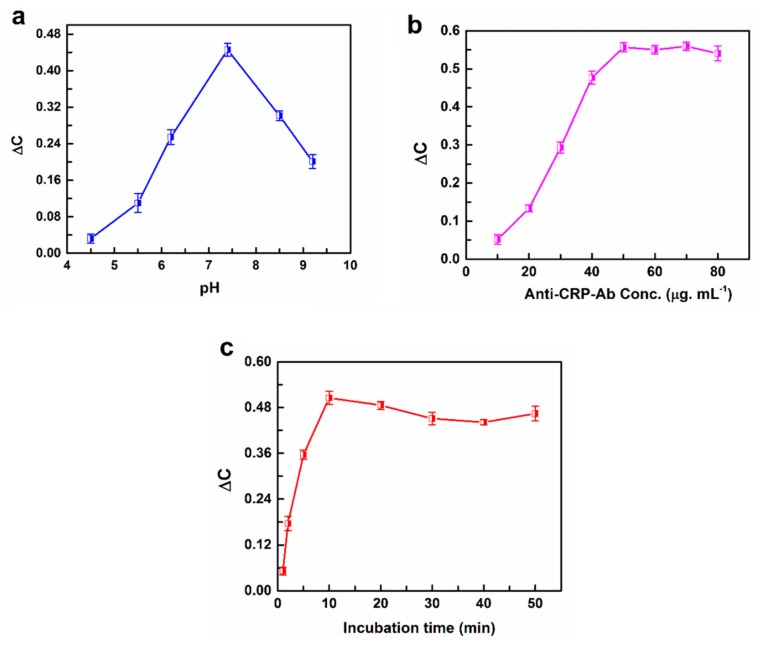
Effect of pH (**a**), concentration of Anti-CRP-Ab (**b**) and the incubation time (**c**) on the response of the immunosensor to 1 ng mL^−1^ of CRP analyte.

To obtain the optimal electrochemical response, the experimental conditions in terms of pH (4.5, 5.5, 6.2, 7.4, 8.5, 9.2), anti-CRP-Ab concentration (10, 20, 30, 40, 50, 60, 70, and 80 µg mL^−1^), and incubation time (1, 2, 5, 10, 20, 30, 40, and 50 min) are optimized, where CRP concentration was kept constant (1 ng mL^−1^). As shown in Figure 4a, the capacitance change increases with increase in pH value until 7.4, and then decreases with the increase in pH value. Thus, the optimal pH for immunoreaction is 7.4, since either extreme alkaline or acidic pH conditions may denature proteins. A suitable concentration of antibody was investigated for the immunocomplex formation with antigen to obtain the best sensor performance. As shown in Figure 4b, the sensor was assessed by incubating in various concentrations of anti-CRP-antibody ranging from 5.0 to 200 µg mL^−1^. The ΔC of the sensor increased as the concentration is increased from 5.0 to 50 µg mL^−1^, while above 50 µg mL^−1^, no considerable increase in the sensor response was detected due to saturation effect. Hence, 50 µg mL^−1^ of anti-CRP-antibody concentration was used for the later experiments. The effect of incubation time between anti-CRP-antibody and CRP was studied and optimized between 1.0 and 50 min. The response signal increased with increase in the immunoreaction time up to 10 min, while above this incubation time, no significant increase in the signal was detected, suggesting that 10 min is the optimum time for the immunoreaction (Figure 4c). To summarize the optimized conditions, the optimum pH value is 7.4, concentration of anti-CRP-antibody is 50 µg mL^−1^ and incubation time is 10 min.

### 3.3. Voltammetry and Impedance Analysis of Modified Electrode Arrays

**Figure 5:**
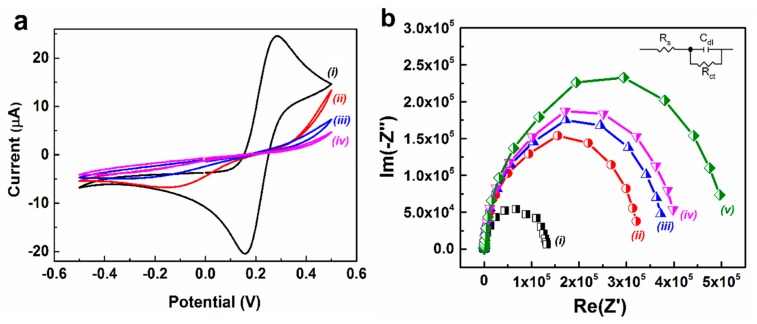
CV (**a**) and Nyquist plot (**b**) of bare IDWµE (*i*), DTSP/IDWµE (*ii*), anti-CRP-antibody/DTSP/IDWµE (*iii*) and BSA/anti-CRP-antibody/DTSP/IDWµE arrays (*iv*) and BSA/anti-CRP-antibody/DTSP/IDWµE arrays with 0.1 ng mL^−^^1^ of CRP (*v*) in 5 mM K_3_Fe(CN)_6_/K_4_Fe(CN)_6_ (1:1) and 0.1 M KCl in 1× PBS.

Figure 5a shows the voltammetry behavior of the step-wise fabrication process for the bare and modified electrodes in a pH 7.4, PBS containing 5 mM [Fe(CN)_6_]^3−^/^4−^) and 0.1 M KCl. A pair of well-defined redox peaks were detected in case of bare electrode suggesting a clear electrochemical response for [Fe(CN)_6_]^3−^/^4−^ with gold surface (Figure 5a, curve *i*). The electrodes modified with functionalized SAM shows a drastic change in voltammetry curves and peak currents, compared to bare electrode (Figure 5a, curve *ii*). This phenomenon may be ascribed to the compact nature of the functionalized SAM coated on the electrode surface which hindered the electron transport or blockage of [Fe(CN)_6_]^3−^/^4−^ ion movements towards the electrode surface. In addition, CV of anti-CRP-antibody and BSA-modified electrodes showed reduced ferricyanide responses in comparison to SAM functionalized electrode arrays (Figure 5a, curve *iii* and *iv*). This phenomenon may arise due to formation of an insulating biomolecular layer of anti-CRP-antibody and BSA on the electrode surface which prevents the transfer of electrons to the electrode surface from the redox electrochemical probe. This implies the complete decrease in peak currents for [Fe(CN)_6_]^3−^/^4−^ redox probe and shifts in reductive peak potential for the anti-CRP-antibody and BSA modified electrode arrays.

EIS was performed to probe the interfacial properties of the electrode surface during electrode modification steps (SAM functionalization and bio-immobilization of anti-CRP-antibody). Figure 5b shows the Nyquist plots (−*Z*′′ vs. *Z*′) obtained on bare and modified IDWµE arrays in a solution of 5 mM K_3_Fe(CN)_6_/K_4_Fe(CN)_6_ (1:1) and 0.1 M KCl and in 1× PBS, respectively. The charge-transfer resistance (*R*_ct_) at the electrode surface was calculated by fitting the measured impedance data to an equivalent circuit model as shown in inset of Figure 5b. The measured impedance of the fabricated electrode sensors might be analyzed by using a simplified Randles equivalent circuit model consists of a series solution resistance (R_S_), electrode double layer capacitance which is in parallel to the charge transfer resistance. As shown from Table S1, it was determined that the *R*_ct_ tends to increase with step-wise assembly of the bare IDWµE and modified IDWµE electrodes. The impedance spectra of the bare IDWµE arrays (Figure 5b, curve *i*) shows a semicircle (*R*_ct_: 123,180 Ω) with no diffusional limiting step of the electrochemical process which is an advantage of the fabricated microelectrodes. The charge transfer resistance of the SAM modified electrode (*R*_ct_: 311540) increases due to the surface functionalization of reduced DTSP layer (Figure 5b, curve *ii*). This suggests that the assembled monolayer generated a hindrance to the electron transfer of [Fe(CN)_6_]^3−^/^4−^) at the electrode surface. After the immobilization of Anti-CRP-antibodies on the DTSP-SAM, the diameter of the high frequency semicircle was significantly enhanced with an *R*_ct_ value of 358,210 Ω (Figure 5b, curve *iii*), suggesting that Anti-CRP-antibodies were covalently immobilized onto the SAM modified electrode arrays. The increase in *R*_ct_ values might be due the presence of biological molecule on the electrode surface which results in formation of a ferricyanide transport-blocking layer on the electrode surface. The modification of electrode with 1 mg mL^−1^ of BSA and introduction of 0.1 ng mL^−1^ of CRP onto the electrode surface increased the *R*_ct_ to 384,650 Ω and 473,850 Ω, respectively (Figure 5b, curve *iv* and *curve* *v*). The increase in biological moieties on the electrode surface obstruct the electron transfer of electrochemical redox probe at the modified electrode surfaces. This implies that the non-specific blocking of modified electrodes and the analyte (CRP) induced response of the fabricated immunosensor. The impedance results are consistent with that of CV experiments as shown in Figure 4a, indicating the functionalization of electrode with DTSP-SAM and successful immobilization of anti-CRP-antibodies on the electrode surface.

**Figure 6:**
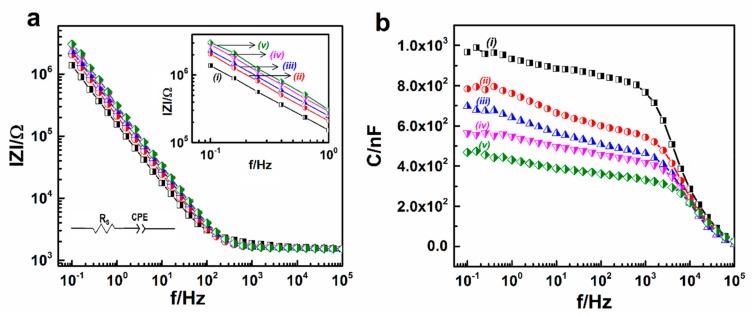
Bode plot of the impedance magnitude (∣Z∣) (**a**) and reactive capacitance (C) (**b**) obtained at IDWµE (*i*), DTSP/IDWµE (*ii*), anti-CRP-antibody/DTSP/IDWµE (*iii*) BSA/anti-CRP-antibody/DTSP/IDWµE arrays (*iv*) and BSA/anti-CRP-antibody/DTSP/IDWµE array with 0.1 ng mL^−^^1^ (*v*) of CRP at various modification stages of the electrode surface for the detection of CRP in 1× PBS.

The impedance magnitudes (∣Z∣) and corresponding capacitances (C) of bare electrode arrays and modified electrode arrays for detection of CRP in 1× PBS are shown in Figure 6a,b. The measured impedance of the fabricated electrode sensors might be analyzed by using an equivalent circuit model consists of an electrode interfacial impedance modeled as the constant phase element (CPE) and a series solution resistance (R_S_) [29]. The impedance of the modified electrodes increased linearly which is typical of the capacitance determined from the following equation of impedance; Z_CPE_ = [*Q*(*jω*)*^n^*]^−1^; where *Q* (CPE-T) and *n* (CPE-P) are numerical values, Z is impedance, *j* is an imaginary unit, and *ω* is the angular frequency (Figure 5a). By using non-linear curve fitting, the values of these parameters were extrapolated to reduce the sum of the squared deviations between the equivalent circuit model and the measured impedance spectra. As shown from Table 1, it was determined that the 1/CPE-T tends to decrease with step-wise assembly and the CPE-P of the bare IDWµE and modified IDWµE electrodes was reached close to 1 after each modification step, suggesting that the interfacial electrode impedance was mainly attributed by capacitive reactance [14]. From Figure 6b, the change in capacitance occurred (C_total_) due to the sequential binding of linker molecules (C_SAM_), and proteins; such as antibodies (C_anti-CRP-Ab_) and antigens (C_CRP_) on the electrode surface which results an increase in impedance magnitude (∣Z∣); and on the other hand a decrease in reactive capacitance, as shown in Figure 6a,b, respectively [15].

### 3.4. Capacitive Analysis of CRP in 1× PBS

**Figure 7:**
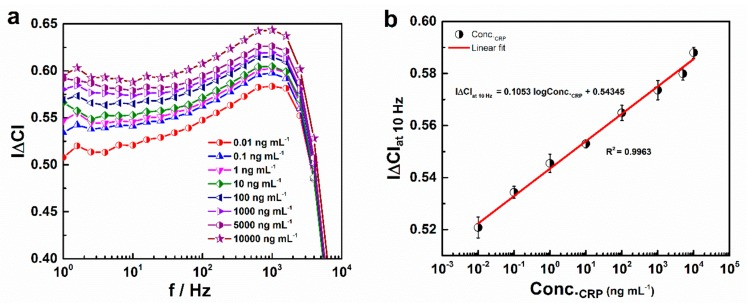
Normalized capacitance (|∆C|) of the BSA/anti-CRP-antibodies/DTSP/IDWµE array measured with respect to increased CRP concentrations diluted in 1× PBS (**a**); Calibration plot for |∆C|_at 10Hz_ with increasing CRP concentrations diluted in 1× PBS and ranging from 0.01 to 10,000 ng mL^−1^ (**b**). Each data point represents the average of three values (*n* = 3), with the range indicated by error bars.

Changes in capacitance (|∆C|) for the BSA/anti-CRP-antibodies/DTSP/IDWµE-based immunosensor with respect to increased CRP concentration from 0.01 to 10,000 ng mL^−1^ diluted in 10 mM PBS (Conc._CRP_) were assessed. With increasing concentrations of CRP antigen, the capacitance was decreased. The immunosensor response to CRP was quantified as capacitance change (|∆C|) given as |*C_i_ − C*_0_/*C*_0_|, where *C_i_* and C_0_ represent the capacitance in the presence and absence of antigen, respectively [30]. The corresponding |∆C| versus frequency (f) curve is presented in Figure 7a. It was demonstrated that CRP can be detected with the fabricated anti-CRP-antibodies/DTSP/IDWµE-based electrode array, where |∆C| is shown as a parameter for quantitative detection of CRP. Figure 6a, shows the maximum difference in |∆C| at 10 Hz; this was selected for assessing sensor performance with respect to increasing CRP antigen concentration. A calibration curve based on the changes in |∆C|_at 10Hz_ with logarithmic concentrations of antigen (Conc._CRP_) in PBS from 0.01 to 10,000 ng mL^−1^ is shown in Figure 7b. The calculated regression equation was found to be *y* = 0.1053*x* + 0.543 (*x*: ng mL^−1^, *y*: |∆C|_at 10Hz_) with R^2^ = 0.9963. By considering the capacitance of the antibody-immobilized IDWµE as a threshold of the signal, a detection limit of 0.025 ng mL^−1^ was calculated using the formula 3 × SE/sensitivity, where SE is the standard error of the Y‒intercept (SE = 8.9039 × 10^−4^) and sensitivity (0.1053 ∆C/ng mL^−1^) is the slope of the calibration curve [31] (Figure 7b).

In addition, the dynamic range and detection limits of the present fabricated CRP immunosensor were significantly improved in comparison to other recently developed CRP immunosensors reported in the literature, as shown in Table 2. The limit of detection in our case is higher than [16,32,33,34,35,36], while lower than [37,38,39]. The detection range of the fabricated CRP immunosensor is lower than [32], but wider as compared to some other reported methods [16,34,35,36,37,38,39]. However, the detection assay (a) is label-free, (b) lacks the Warburg diffusion limitation, which may arise from micro-structured fabricated electrodes, (c) has low-cost sample preparation, and (d) is a direct signal readout in the form of an electrical signal, which makes the present fabricated immunosensor advantageous than the reported methods [34,35,36,37,38].

### 3.5. Stability, Reproducibility, and Interference Studies

**Figure 8:**
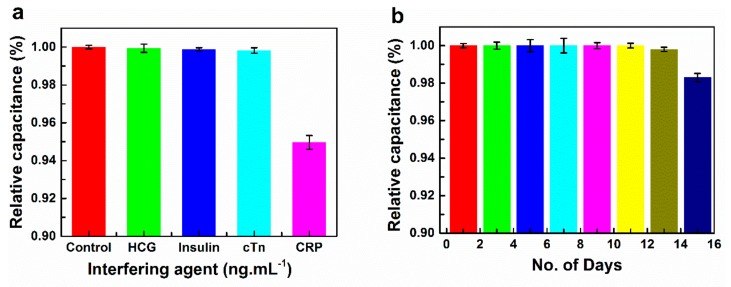
Interference study of the BSA/anti-CRP-antibodies/DTSP/IDWµE array with different interfering agents (Human chorionic gonadotrophin (HCG); Insulin; Cardiac troponin-I (cTn)) in 1× PBS, pH 7.4 at a concentration of 0.1 ng mL^−1^ and a frequency of 10 Hz (**a**); Storage stability study of the BSA/anti-CRP-antibodies/DTSP/IDWµE array at a regular interval of 1 day in 100 µL of 1× PBS (pH 7.4) at 0.1 ng mL^−1^ and a frequency of 10 Hz (**b**). Each data point represents the average of three values (*n* = 3), with the range indicated by error bars.

The variation in capacitance after CRP addition was measured as a function of time in days using as prepared BSA/anti-CRP-antibodies/DTSP/IDWµE biosensor. The storage stability of biosensor was determined by measuring capacitance with 0.1 ng mL^−1^ CRP at regular intervals of 1 day for approximately 2 weeks. The biosensor was stored at 4 °C when not in use. The results demonstrated that the immunosensor lost 3% of its initial response after 2 weeks when stored at 4 °C (Figure 8b). Reproducibility is an important factor for the clinical use of fabricated immunosensors. Reproducibility of our immunosensor was verified by determination of intra- and inter-assay relative standard deviation (RSD). The intra-assay precision of the immunosensor was assessed at 0.1 ng mL^−1^ CRP concentration with three replicate measurements. The inter-assay precision was assessed at 0.1 ng mL^−1^ CRP concentration with three different BSA/anti-CRP-antibodies/DTSP/IDWµE arrays. The calculated intra-assay and inter assay RSDs were 1.95% and 1.70%, respectively, indicating satisfactory precision and reproducibility of the immunosensor. The covalent cross-linking of the anti-CRP-antibody molecules with the electrode surface prevents the escape of these biomolecules from the electrode surface, endorsing good electrode stability. Effects of interferents in CRP detection were investigated using human chorionic gonadotrophin (HCG) (0.1 ng mL^−1^), insulin (0.1 ng mL^−1^), and cardiac troponin (cTn-I) (0.1 ng mL^−1^) and the prepared BSA/anti-CRP-antibodies/DTSP/IDWµE array sensor. The interferents in 1× PBS were incubated with the biosensor separately, and the capacitance was measured over the frequency range 1 Hz to 100,000 Hz. The results suggested that capacitance of the immunosensor was not affected significantly in the presence of selected interfering agents (Figure 8a), compared to control electrodes, demonstrating that the fabricated immunosensor showed high selectivity for the detection of CRP.

### 3.6. Capacitive Detection of CRP in Human Serum

**Figure 9:**
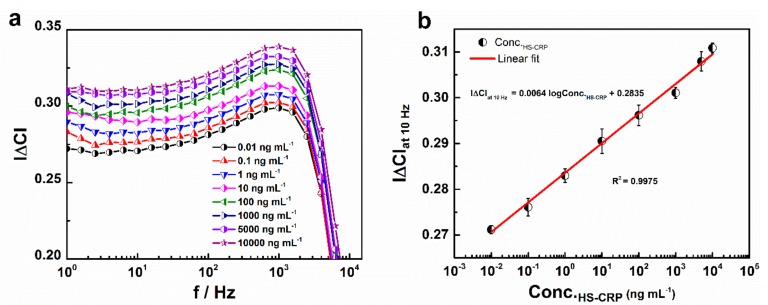
Normalized capacitance (|∆C|) of the BSA/anti-CRP-antibodies/DTSP/IDWµE array measured with respect to increasing CRP concentrations diluted in HS (Conc._HS-CRP_) (**a**); calibration plot for |∆C|_at 10Hz_ with increasing CRP concentrations diluted in HS ranging from 0.01 to 10,000 ng mL^−1^ (**b**). Each data point represents the average of three values (*n* = 3), with the range indicated by error bars.

To validate the practical application of the fabricated immunosensor, the BSA/anti-CRP-antibodies/DTSP/IDWµE arrays were incubated with different concentrations of CRP (0.01 to 10,000 ng mL^−1^) prepared in 1% *v/v* HS samples. From the |∆C| versus f plot, as shown in Figure 9a, it was shown that |∆C| increased with the concentration of CRP in HS. The maximum change in |∆C| was observed at 10 Hz, and therefore, |∆C|_at 10Hz_ was plotted against the various concentrations of CRP in HS (Conc._HS-CRP_). The increase in capacitance with logarithmic CRP concentrations between 0.01 and 10,000 ng mL^−1^ showed a linear relationship with the regression equation as *y* = 0.0064*x* + 0. 2835 (*x*: ng mL^−1^, *y*: |∆C|_at 10Hz_) and R^2^ = 0.9975. The detection limit of 0.23 ng mL^−1^ was calculated using the formula 3 × SE/sensitivity, where SE is the standard error of the Y‒intercept (SE = 4.977 × 10^−4^) and sensitivity (0.0064 ∆C/ng mL^−1^) is the slope of the calibration curve [31] (Figure 9b).

### 3.7. Real Sample Analysis

To validate the capacitance immunosensor, the fabricated BSA/anti-CRP-antibodies/DTSP/IDWµE array was used for the determination of CRP in real samples. Five different concentrations of CRP, 0.5, 250, 500, 1000, and 5000 ng mL^−1^ were spiked on 1% *v/v* PBS diluted serum sample and was applied to the BSA/anti-CRP-antibodies/DTSP/IDWµE array in 1× PBS, pH 7.4. Triplicate of each concentration was tested and the average of three determinants was taken and the recovery was tabulated. From Table 3, concentration of CRP in spiked human serum samples were found to be 0.489, 249.51, 505.42, 998.57, and 4999.13 ng mL^−1^, and the recoveries were 97.8% (n = 3), 99.8% (n = 3), 101.1% (n = 3), 99.8 (n = 3), and 99.9 (n = 3), respectively. The results obtained are in the good range of recovery for three determinants and there was no significant difference between the spiked and the found CRP concentrations using the fabricated immunosensor. Therefore, BSA/anti-CRP-antibodies/DTSP/IDWµE array can be successfully applied for the precise determination of CRP in real samples.

## 4. Conclusions

An electrochemical disposable immunosensor based on capacitance measurement for CRP detection was fabricated by the covalent crosslinking of anti-CRP-antibodies onto a DTSP-SAM-functionalized IDWµE. The functionalization of SAM on the IDWµE surface was established based on AFM, EDX, XPS, and EIS analyses. A biocompatible environment was induced by functionalized SAM to immobilize the anti-CRP-antibodies, which maintained the biological activity of the antibodies. The covalent crosslinking of the antibody to the gold surface, which eliminated additional activation steps (EDC/NHS), afforded good stability to the immunosensor. The immunosensor is optimized for the critical parameters such as pH, anti-CRP-antibody concentration and immunoreaction time. The biosensor was also successfully validated for use as a capacitive immunosensor for the determination of CRP levels in HS samples. The immunosensor exhibited a detection limit of 0.025 ng mL^−1^ and 0.23 pg mL^−1^ in PBS and HS, respectively. The sensor showed a wide dynamic range of 0.01 to 10,000 ng mL^−1^ in both PBS and HS, respectively. The CRP spiked diluted human serum (1% *v*/*v*) sample had shown the recoveries ranging from 97.8–101.1%. Stability studies endorse that the fabricated electrochemical immunosensor has good storage stability. Moreover, interfering agents at higher concentrations have no significant effect on the capacitive detection of CRP at BSA/anti-CRP-antibodies/DTSP/IDWµE array. The miniaturized design of the wave-shaped electrode array and capability of the sensor to incorporate a potentiostat render its applicability for point of care testing in the near future. The fabricated immunosensor could be successfully applied for disease diagnosis in suspected individuals, with higher fold of CRP concentrations (10,000 ng mL^−1^).

## Figures and Tables

**Table 1:** Extrapolated electrochemical impedance spectroscopy (EIS) parameters of the bare IDWµE and modified IDWµE arrays by fitting to the experimental data in Figure 6a.

		CPE		
Electrode	*R_s_* (Ω)	*Q* [×10^−7^·Ω^−1^·s^n^]	*n*	*χ* ^2^
IDWµE array	1493	33.87	0.8569	0.0059
DTSP-SAM	1547	9.641	0.9429	0.0057
Anti-CRP-Ab	1531	6.079	0.9284	0.0051
BSA	1530	6.809	0.9498	0.0048

**Table 2:** Comparison of the performance of our fabricated CRP immunosensor with other reported microsystems for CRP detection.

Assay Type	Detection Limit (ng mL^−1^)	Dynamic Range (ng mL^−1^)	Reference
Paper based EIA	15	50–10^5^	[32]
Immunochromatography	600	N.R	[33]
Impedimetric IA	0.06	1–1000	[16]
Smart phone based IA	0.07	0.03–81	[34]
LOC-based CIA	1.5	3–81	[35]
EIS	0.1	0.1–20	[36]
LSPR	10^−9^	10^−7^–1000	[37]
ETC	5 × 10^−6^	0.01–10	[38]
Piezoelectric IA	3 × 10^−4^	0.001–100	[39]
Impedimetric IA	0.025	0.01–10,000	This work (PBS)
Impedimetric IA	0.23	0.01–10,000	This work (Human-serum)

N.R: not reported, LOC: lab-on-chip, CIA: chemiluminescence immunoassay, EIA: electrochemical immunoassay, ETC: electronic taste chip, LSPR: localized surface plasmon resonance.

**Table 3:** Recovery and % RSD of CRP spiked diluted human serum samples.

Test Sample	CRP in Diluted Serum (ng mL^−1^)	Spiked (ng mL^−1^)	Found (ng mL^−1^)	Recovery (%)	RSD (%)
A	0	0.5	0.489	97.8	1.52
B	0	250	249.51	99.8	1.99
C	0	500	505.72	101.1	1.25
D	0	1000	998.57	99.8	1.90
E	0	5000	4999.13	99.9	1.32

## Supplementary Materials

The following are available online at https://www.mdpi.com/1424-8220/19/24/5560/s1, Table S1: Extrapolated electrochemical impedance spectroscopy parameters of the bare IDWµE and modified IDWµE arrays by fitting the experimental data in Figure 5b to a simplified Randles equation; Figure S1: Photograph of the Interdigitated wave type microelectrode array on a slide glass substrate (63 × 16 × 1.1 mm in thickness) with conductive pads and sensing area (30 µm spacing and width) (**a**), Mounting of a polydimethylsiloxane chamber (d = 8 mm) prepared by a standard protocol (10:1) on the sensing area to assist with the SAM functionalization and to preserve the solvents for immunoreaction (**b**), Giving an external connection to the conductive pads using silver conductive epoxy adhesive (**c**); Figure S2: EDX elemental analysis of the Interdigitated wave shaped microelectrode array before (**a**) and after SAM treatment (**b**).

## References

[1] Kim K.W., Kim B., Moon H.W., Lee S.H., Kim H.R. (2015). Role of C-reactive protein in osteoclastogenesis in rheumatoid arthritis. Arthritis Res. Ther..

[2] Shrotriya S., Walsh D., Bennani-Baiti N., Thomas S., Lorton C. (2015). C-Reactive Protein Is an Important Biomarker for Prognosis Tumor Recurrence and Treatment Response in Adult Solid Tumors: A Systematic Review. PLoS ONE.

[3] Thangamuthu M., Santschi C., Martin O.J.F. (2018). Label-Free Electrochemical Immunoassay for C-Reactive Protein. Biosensors.

[4] Johnson A., Song Q., Ferrigno P.K., Bueno P.R., Davis J.J. (2012). Sensitive Affimer and Antibody Based Impedimetric Label-Free Assays for C-Reactive Protein. Anal. Chem..

[5] Pepys M.B., Hirschfield G.M. (2003). C-reactive protein: A critical update. J. Clin. Investig..

[6] Choi Y.H., Ko H., Lee G.Y., Chang S.Y., Chang Y.W., Kang M.J. (2015). Development of a sensitive SPR biosensor for C-reactive protein (CRP) using plasma-treated Parylene-N film. Sens. Actuators B Chem..

[7] Yen Y.K., Lai Y.C., Hong W.T., Pheanpanitporn Y., Chen C.S., Huang L.S. (2013). Electrical Detection of C-Reactive Protein Using a Single Free-Standing, Thermally Controlled Piezoresistive Microcantilever for Highly Reproducible and Accurate Measurements. Sensors.

[8] Wu J.G., Wei S.C., Chen Y., Chen J.H., Luo S.C. (2018). Critical Study of the Recognition between C-Reactive Protein and Surface-Immobilized Phosphorylcholine by Quartz Crystal Microbalance with Dissipation. Langmuir.

[9] Jampasa S., Siangproh W., Laocharoensuk R., Vilaivan T., Chailapakul O. (2018). Electrochemical detection of C-reactive protein based on anthraquinone labeled antibody using a screen-printed graphene electrode. Talanta.

[10] Vashist S.K., Venkatesh A.G., Schneider E.M., Beaudoin C., Luppa P.B., Luong J.H.T. (2016). Bioanalytical advances in assays for C-reactive protein. Biotechnol. Adv..

[11] Ledue T.B., Rifai N. (2001). High sensitivity immunoassays for C-reactive protein: Promises and pitfalls. Clin. Chem. Lab. Med..

[12] Fakanya W.M., Tothill I.E. (2014). Detection of the Inflammation Biomarker C-Reactive Protein in Serum Samples: Towards an Optimal Biosensor Formula. Biosensors.

[13] Kitayama Y., Takeuchi T. (2014). Localized surface Plasmon resonance nanosensing of C reactive protein with poly(2-methacryloyloxyethyl phosphorylcholine)-grafted gold nanoparticles prepared by surface-initiated atom transfer radical polymerization. Anal. Chem..

[14] Yagati A.K., Park J., Kim J., Ju H., Chang K.-A., Cho S. (2016). Sensitivity enhancement of capacitive tumor necrosis factor-α detection. Jpn. J. Appl. Phys..

[15] Yagati A.K., Park J., Cho S. (2016). Reduced Graphene Oxide Modified the Interdigitated Chain Electrode for an Insulin Sensor. Sensors.

[16] Yagati A.K., Pyun J.C., Min J., Cho S. (2016). Label-free and direct detection of C-reactive protein using reduced graphene oxide-nanoparticle hybrid impedimetric sensor. Bioelectrochemistry.

[17] Fernandes F.C., Santos A., Martins D.C., Goes M.S., Bueno P.R. (2014). Comparing label free electrochemical impedimetric and capacitive biosensing architectures. Biosens. Bioelectron..

[18] Soares A.C., Soares J.C., Shimizu F.M., Rodrigues V.D.C., Awan I.T., Melendez M.E., Piazzetta M.H.O., Gobbi R.M., Reis A.L., Fregnani J.H.T.G. (2018). A simple architecture with self-assembled monolayers to build immunosensors for detecting the pancreatic cancer biomarker CA19-9. Analyst.

[19] Muharemagic D., Labib M., Ghobadloo S.M., Zamay A.S., Bell J.C., Berezovski M.V. (2012). Anti-Fab Aptamers for Shielding Virus from Neutralizing Antibodies. J. Am. Chem. Soc..

[20] Limbut W., Kanatharana P., Mattiasson B., Asawatreratanakul P., Thavarungkul P. (2006). A comparative study of capacitive immunosensors based on self-assembled monolayers formed from thiourea, thioctic acid, and 3-mercaptopropionic acid. Biosens. Bioelectron..

[21] Zhu N., Ulstrup J., Chi Q. (2015). Surface self-assembled hybrid nanocomposites with electroactive nanoparticles and enzymes confined in a polymer matrix for controlled electrocatalysis. J. Mater. Chem. B.

[22] Longo E., Wright K., Caruso M., Gatto E., Palleschi A., Scarselli M., De Crescenzi M., Crisma M., Formaggio F., Toniolo C. (2015). Peptide flatlandia: A new-concept peptide for positioning of electroactive probes in proximity to a metal surface. Nanoscale.

[23] Kaushik A., Yndart A., Kumar S., Jayant R.D., Vashist A., Brown A.N., Li C.-Z., Nair M. (2018). A sensitive electrochemical immunosensor for label-free detection of Zika-virus protein. Sci. Rep..

[24] Arya S.K., Chornokur G., Venugopal M., Bhansali S. (2010). Dithiobis(succinimidyl propionate) modified gold microarray electrode based electrochemical immunosensor for ultrasensitive detection of cortisol. Biosens. Bioelectron..

[25] Pasha S.K., Kaushik A., Vasudev A., Snipes S.A., Bhansali S. (2014). Electrochemical immunosensing of saliva cortisol. J. Electrochem. Soci..

[26] Jia J., Kara A., Pasquali L., Bendounan A., Sirotti F., Esaulov V.A. (2015). On sulfur core level binding energies in thiol self-assembly and alternative adsorption sites: An experimental and theoretical study. J. Chem. Phys..

[27] Willey T.M., Vance A.L., Bostedt C., van Buuren T., Meulenberg R.W., Terminello L.J., Fadley C.S. (2004). Surface Structure and Chemical Switching of Thioctic Acid Adsorbed on Au (111) As Observed Using Near-Edge X-ray Absorption Fine Structure. Langmuir.

[28] Mazzotta E., Rella S., Turco A., Malitesta C. (2015). XPS in development of chemical sensors. RSC. Adv..

[29] Shoar Abouzari M.R., Berkemeier F., Schmitz G., Wilmer D. (2009). On the physical interpretation of constant phase elements. Solid. State. Ion..

[30] Yagati A.K., Choi Y., Park J., Choi J.-W., Jun H.-S., Cho S. (2016). Silver nanoflower-reduced graphene oxide composite based micro-disk electrode for insulin detection in serum. Biosens. Bioelectron..

[31] Gobi K.V., Iwasaka H., Miura N. (2007). Self-assembled PEG monolayer based SPR immunosensor for label-free detection of insulin. Biosens. Bioelectron..

[32] Boonkaew S., Chaiyo S., Jampasa S., Rengpipat S., Siangproh W., Chailapakul O. (2019). An origami paper-based electrochemical immunoassay for the C-reactive protein using a screen-printed carbon electrode modified with graphene and gold nanoparticles. Microchim. Acta.

[33] Byzova N.A., Zherdev A.V., Vengerov Y.Y., Starovoitova T.A., Dzantiev B.B. (2017). A triple immunochromatographic test for simultaneous determination of cardiac troponin I, fatty acid binding protein and C-reactive protein biomarkers. Microchim. Acta.

[34] Vashist S.K., Schneider E.M., Zengerle R., Stetten F.V., Luong J.H.T. (2015). Graphene-based rapid and highly-sensitive immunoassay for C-reactive protein using a smartphone-based colorimetric reader. Biosens. Bioelectron..

[35] Czilwik G., Vashist S.K., Klein V., Buderer A., Roth G., Stetten F.V. (2015). Magnetic chemiluminescent immunoassay for human C-reactive protein on the centrifugal microfluidics platform. RSC Adv..

[36] Chen X., Wang Y., Zhou J., Yan W., Li X., Zhu J. (2008). Electrochemical impedance immunosensor based on three-dimensionally ordered macroporous gold film. Anal. Chem..

[37] Yeom S.-H., Han M.-E., Kang B.-H., Kim K.-J., Yuan H., Eum N.-S., Kang S.-W. (2013). Enhancement of the sensitivity of LSPR-based CRP immunosensors by Au nanoparticle antibody conjugation. Sens. Actuators B Chem..

[38] Christodoulides N., Mohanty S., Miller C.S., Langub M.C., Floriano P.N., Dharshan P., Ali M.F., Bernard B., Romanovicz D., Anslyn E. (2005). Application of microchip assay system for the measurement of C-reactive protein in human saliva. Lab Chip.

[39] Zhou J., Gan N., Li T., Zhou H., Li X., Cao Y., Wang L., Sang W., Hu F. (2013). Ultratrace detection of C-reactive protein by a piezoelectric immunosensor based on Fe_3_O_4_@SiO_2_ magnetic capture nanoprobes and HRP-antibody co-immobilized nano gold as signal tags. Sens. Actuators B Chem..

