# Supplementary material for: Electrochemical Detection of C-Reactive Protein in Human Serum Based on Self-Assembled Monolayer-Modified Interdigitated Wave-Shaped Electrode
Source: Sensors (Basel). 2019 Dec 16;19(24):5560. doi: 10.3390/s19245560 (PMC6960938; doi:10.3390/s19245560)
Supplement: Supplementary file 1 [file sensors-19-05560-s001.pdf]

Supplementary Material

Electrochemical Detection of C-Reactive Protein in Human Serum Based on Self-Assembled Monolayer-Modified Interdigitated Wave-Shaped Electrode

Somasekhar R. Chinnadayyala [1], Jinsoo Park [2], Young Hyo Kim [3], Seong Hye Choi [4], Sang-Myung Lee [5], Won Woo Cho [6], Ga-Yeon Lee [7], Jae-Chul Pyun [7] and Sungbo Cho [1,2,*]

1. Department of Electronic Engineering, Gachon University, Seongnam-si, Gyeonggi-do, Incheon 13120, Korea; ssreddy@gachon.ac.kr
2. Gachon Advanced Institute for Health Science & Technology, Gachon University, Incheon 21999, Korea; jspark88@gc.gachon.ac.kr
3. Department of Otorhinolaryngology-Head and Neck Surgery, School of Medicine, Inha University, Incheon 22332, Korea; inhaorl@inha.ac.kr
4. Department of Neurology, School of Medicine, Inha University, Incheon 22332, Korea; seonghye@inha.ac.kr
5. Department of Chemical Engineering, Kangwon National University, Chuncheon 25341, Korea; sangmyung@kangwon.ac.kr
6. Cantis Inc., Ansan-si, Gyeonggi-do 15588, Korea; wwcho@cantis.co.kr
7. Department of Materials Science and Engineering, Yonsei University, Seoul 03772, Korea; gayeon@yonsei.ac.kr (G.-Y.L.); jcpyun@yonsei.ac.kr (J.-C.P.)

* Correspondence: sbcho@gachon.ac.kr; Tel.: +82-31-750-5321

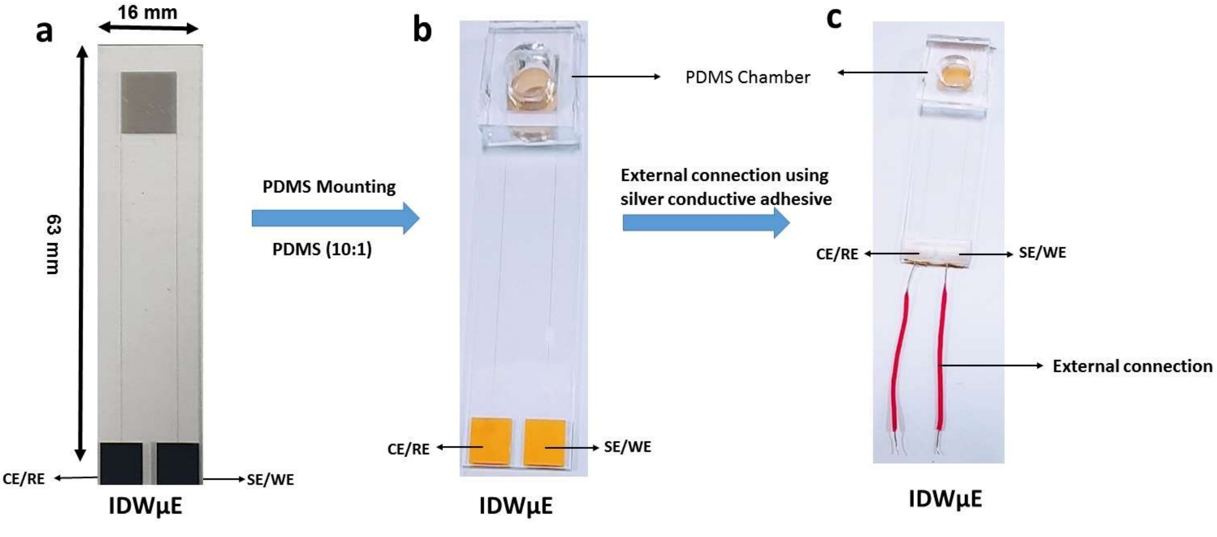

Figure. S1. Photograph of the Interdigitated wave type microelectrode array on a slide glass substrate (63 × 16 × 1.1 mm in thickness) with conductive pads and sensing area (30 μm spacing and width) (**a**), Mounting of a polydimethylsiloxane chamber (d = 8 mm) prepared by a standard protocol (10:1) on the sensing area to assist with the SAM functionalization and to preserve the solvents for immunoreaction (**b**), Giving an external connection to the conductive pads using silver conductive epoxy adhesive (**c**).

(a)

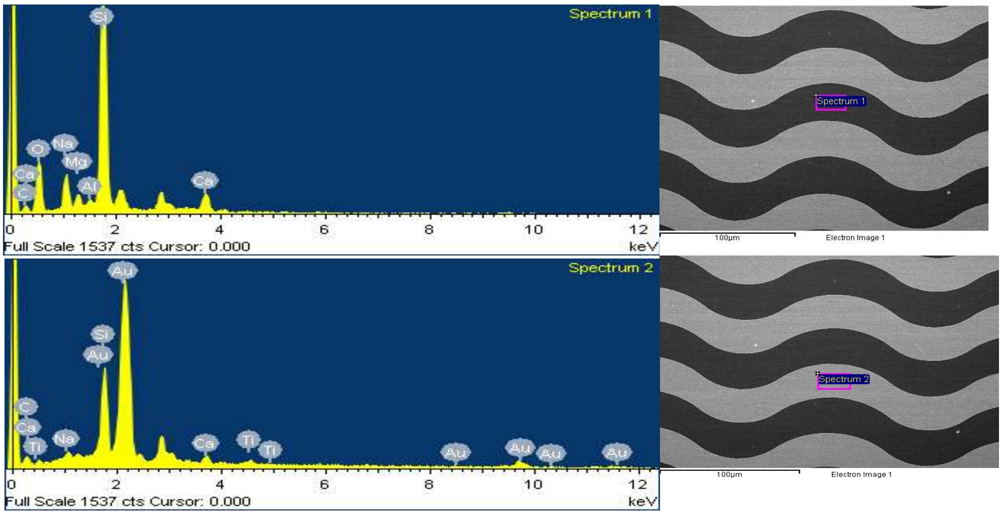

(b)

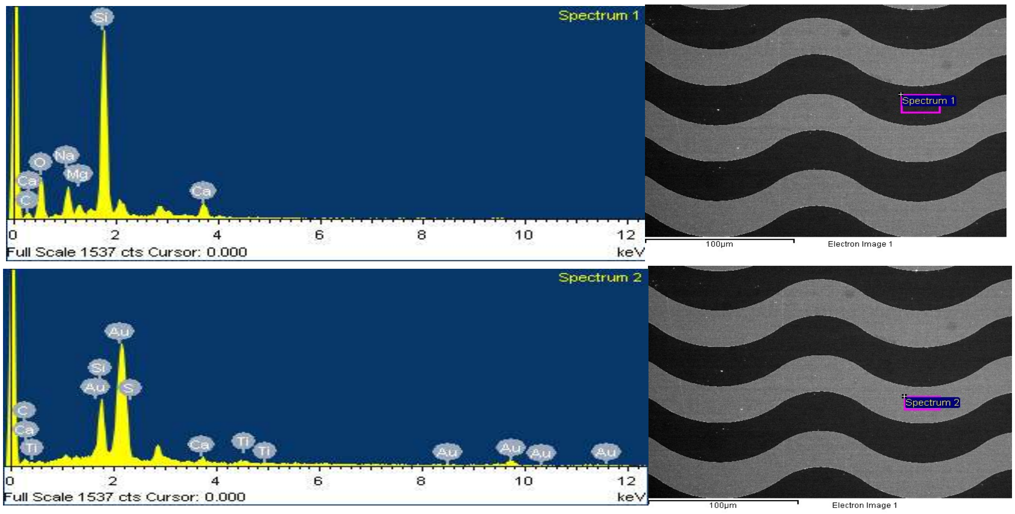

Figure. S2. EDX elemental analysis of the Interdigitated wave shaped microelectrode array before (**a**) and after SAM treatment (**b**).

EDX of the electrode array before SAM coating, the spacing area shows Si and O as main elements and C, Al, Ca as trace elements, whereas gold working electrode area shows Au as main element, and Si, Ti, C, Na, Ca as trace elements. After SAM coating on the electrode array, the gold working electrode area shows Au as main element and S, Si, Ti, C, Ca as trace elements, where Au, Si, Ti, stems from electrode surface and S, C stems from SAM coated layer on the electrode array.

Table S1. Extrapolated electrochemical impedance spectroscopy parameters of the bare IDWμE and modified IDWμE arrays by fitting the experimental data in Figure 5b to a simplified Randles equation.

**Electrode**	R_s **(Ω)**	C_{dl}	R_{ct} **(Ω)**	χ^2
IDWμE array	452.7 ± 10	4.06×10^{-1}	123,180 ± 25	0.0016
DTSP-SAM	446.2 ± 2	4.66×10^{-8}	311,540 ± 41	0.0097
Anti-CRP-Ab	455.0 ± 5	4.07×10^{-8}	358,210 ± 36	0.0017
BSA	455.1 ± 4	4.08×10^{-8}	384,650 ± 11	0.0055
CRP (0.1 ng mL^{-1})	499.0 ± 2	3.39×10^{-8}	473,850 ± 28	0.0041

Fabrication of IDWμE Array:

For the fabrication of the IDWμE, a slide glass substrate which has 63 mm in length, 16 mm in width, 1.1 mm in thickness was cleaned by sonication in 70% methanol for 5 minutes. The negative photoresist (DNR L-300-30, Dongjin Semichem, Hwaseong, Korea) was spin-coated at 3000 rpm for 30 sec. to form the photoresist with a thickness of 3 μm on the glass substrate. Subsequently, the photoresist was soft baked at 95 °C for 2 min. for the dehydration of the photoresist solvent. To pattern the IDWμE which has 30 μm of spacing and electrode gap, the ultra-violet (UV) exposure (4.5A, 200 W) was carried out for 18 s by using a mask aligner (MDA-400M, Midas System, Daejeon, Korea). After the post exposure bake (PEB) at 110 °C for 3 min., the substrate was immersed in the developing solution (AZ MIF-300, AZ Electronic Materials, Sommerville, NJ, USA) for 30 sec. and washed with distilled water (DW). After removing the remaining moisture using the N2 gas blowing, the Ti (25 nm) and Au (50 nm) was deposited by using the e-beam evaporator, respectively. Finally, the ICE was obtained from the lift-off process using the acetone with ultra-sonication.
